# Hawksbill turtle terra incognita: conservation genetics of eastern Pacific rookeries

**DOI:** 10.1002/ece3.1897

**Published:** 2016-01-28

**Authors:** Alexander R. Gaos, Rebecca L. Lewison, Michael J. Liles, Velkiss Gadea, Eduardo Altamirano, Ana V. Henríquez, Perla Torres, José Urteaga, Felipe Vallejo, Andres Baquero, Carolina LeMarie, Juan Pablo Muñoz, Jaime A. Chaves, Catherine E. Hart, Alejandro Peña de Niz, Didiher Chácon, Luis Fonseca, Sarah Otterstrom, Ingrid L. Yañez, Erin L. LaCasella, Amy Frey, Michael P. Jensen, Peter H. Dutton

**Affiliations:** ^1^ San Diego State UniversitySan DiegoCalifornia; ^2^ University of California DavisDavisCalifornia; ^3^ Ocean Associates Incunder contract to the Southwest Fisheries Science CenterNational Marine Fisheries ServiceNational Oceanic and Atmospheric AdministrationLa JollaCalifornia; ^4^ Texas A&M UniversityCollege StationTexas; ^5^ Fauna & Flora InternationalManaguaNicaragua; ^6^ Stanford UniversityPalo AltoCalifornia; ^7^ Fundación Equilibrio AzulQuitoEcuador; ^8^ Universidad San Francisco de Quito‐Galapagos Science CenterIsla San CristobalEcuador; ^9^ Red Tortuguera, A.C.GuayabitosNayaritMexico; ^10^ Grupo Tortuguero de las Californias, A.C.La PazBaja California SurMexico; ^11^ Marine Turtle Research GroupCornwall CampusCenter for Ecology and ConservationSchool of BiosciencesUniversity of Exeter Cornwall CampusPenrynCornwallU.K; ^12^ Centro de Protección y Conservación de Tortugas Marinas Playa TeopaJaliscoMexico; ^13^ Latin American Sea TurtlesTibasSan JoseCosta Rica; ^14^ Paso PacificoManaguaNicaragua; ^15^ Southwest Fisheries Science CenterNational Marine Fisheries ServiceNational Oceanic and Atmospheric AdministrationLa JollaCalifornia; ^16^ Eastern Pacific Hawksbill InitiativeSan DiegoCalifornia

**Keywords:** Critically endangered, *Eretmochelys imbricata*, management units, mangroves, phylogeography, reproductive ecotype

## Abstract

Prior to 2008 and the discovery of several important hawksbill turtle (*Eretmochelys imbricata*) nesting colonies in the EP (Eastern Pacific), the species was considered virtually absent from the region. Research since that time has yielded new insights into EP hawksbills, salient among them being the use of mangrove estuaries for nesting. These recent revelations have raised interest in the genetic characterization of hawksbills in the EP, studies of which have remained lacking to date. Between 2008 and 2014, we collected tissue samples from 269 nesting hawksbills at nine rookeries across the EP and used mitochondrial DNA sequences (766 bp) to generate the first genetic characterization of rookeries in the region. Our results inform genetic diversity, population differentiation, and phylogeography of the species. Hawksbills in the EP demonstrate low genetic diversity: We identified a total of only seven haplotypes across the region, including five new and two previously identified nesting haplotypes (pooled frequencies of 58.4% and 41.6%, respectively), the former only evident in Central American rookeries. Despite low genetic diversity, we found strong stock structure between the four principal rookeries, suggesting the existence of multiple populations and warranting their recognition as distinct management units. Furthermore, haplotypes EiIP106 and EiIP108 are unique to hawksbills that nest in mangrove estuaries, a behavior found only in hawksbills along Pacific Central America. The detected genetic differentiation supports the existence of a novel mangrove estuary “reproductive ecotype” that may warrant additional conservation attention. From a phylogeographic perspective, our research indicates hawksbills colonized the EP via the Indo‐Pacific, and do not represent relict populations isolated from the Atlantic by the rising of the Panama Isthmus. Low overall genetic diversity in the EP is likely the combined result of few rookeries, extremely small reproductive populations and evolutionarily recent colonization events. Additional research with larger sample sizes and variable markers will help further genetic understanding of hawksbill turtles in the EP.

## Introduction

Resolving ecological uncertainties about small and highly endangered wildlife populations is fundamental to their management and recovery (Lande 1988; Pukazhenthi et al. 2005). A multitude of tools (e.g., satellite telemetry, genetics) can be implemented to understand various aspects of wildlife ecology and life history in order to fill existing data gaps, yet generating information on rare species is innately difficult, particularly for highly vagile marine organisms that can be notoriously difficult to locate and study (Costello et al. 2010; Jacobson et al. 2010).

**Figure 1:**
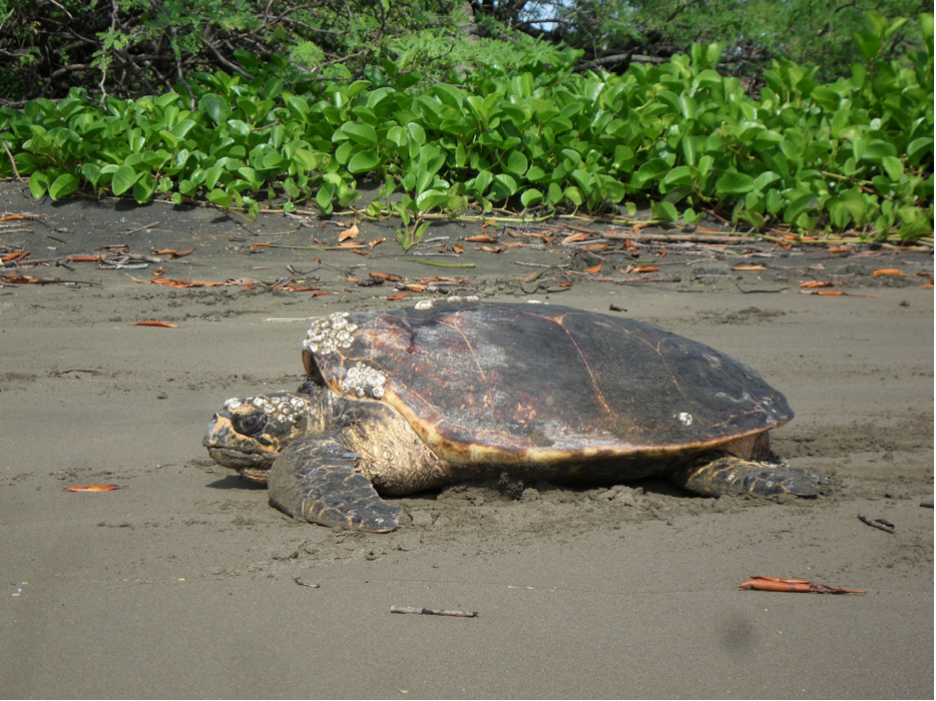
Postnesting hawksbill turtle returning to the estuary in the Estero Padre Ramos Nature Reserve, Nicaragua.

Until 2008 hawksbill turtles (*Eretmochelys imbricata*, Fig. 1) were considered virtually absent from the EP (Eastern Pacific) Ocean (Cornelius 1982; Mortimer and Donnelly 2008). Recent discoveries of several important rookeries spanning from Mexico to Ecuador have improved the species' prognosis (Gaos et al. 2010; Liles et al. 2015), yet hawksbills in the EP are still collectively cited as one of the most endangered marine turtle populations on the planet (Wallace et al. 2011). Although hawksbills were historically much more abundant in the EP, evidence suggests they have always been rarer than the other marine turtle species in the region (Cornelius 1982; Seminoff et al. 2003; Gaos et al. 2010). This is supported by the general absence of historical accounts of hawksbills and is consistent with the limited number of coral reefs in the EP (Glynn 1997; Gaos and Yañez 2012; Gaos et al. 2012a,b; Seminoff et al. 2012), which is considered the primary habitat for the species in most ocean regions (Meylan 1988; Limpus 1992; Leon and Bjorndal 2002).

Since the extant rookeries were identified in the EP, several research and conservation projects have been established. One of the most important findings to date has been that in contrast to their Atlantic and Indo‐Pacific counterparts, where hawksbills associate with coral reefs, adult hawksbills in the EP primarily use mangrove estuaries for nesting and foraging (Gaos et al. 2010, 2012a,b; Liles et al. 2015). This is the case for both of the major rookeries located at Bahía de Jiquilisco in El Salvador and Estero Padre Ramos in Nicaragua, which together account for approximately 75% of the nesting in the region (Gaos et al. 2010; Altamirano 2014; Liles et al. 2015). The predominant use of mangrove estuaries for nesting appears to be a behavior unique to hawksbills in the EP and more specifically, in Central America (Gaos et al. 2012a,b; Liles et al. 2015).

From a global phylogeographic perspective, the hawksbill turtle has been shown to have two distinct evolutionary lineages, with a deep bifurcation between those in the Atlantic and the Indo‐Pacific (Okayama et al. 1999; Nishizawa et al. 2010). However, previous studies have omitted hawksbills inhabiting the EP, leaving a significant gap in the phylogenetic characterization of the species. Interest in evaluating genetic stock structure and resolving current phylogeographic relationships of hawksbills in the EP is heightened in light of the unique behavioral and biological traits that characterize individuals in the region, including the aforementioned predominant use of mangrove estuary habitats for nesting and foraging, as well as extremely limited home ranges and postnesting migrations (Gaos and Yañez 2012; Gaos et al. 2012a,b; Liles et al. 2015). Current data gaps hinder our understanding of hawksbill life history, as well as effective management and conservation (Gaos et al. 2012c; Liles et al. 2015).

The use of molecular genetic techniques is a critical tool for understanding and managing at‐risk populations of a wide variety of marine species (Arif et al. 2011), from mammals to invertebrates (Baums 2008; Sanford and Kelly 2011; Oliveira et al. 2012). For marine turtles, genetic studies have supported research into fundamental ecological concepts such as population structure, mating systems, connectivity, historical population trends and phylogeography (Jensen et al. 2013). In turn, this information can be used to inform management, prioritize the allocation of limited resources for conservation (Carvalho and Hauser 1994; Wan et al. 2004), and support governance on scales ranging from the waters of a single nation (e.g., Jensen et al. 2013) to entire ocean basins (e.g., Dutton et al. 1999).

Due to strong philopatry demonstrated by females of the taxon, matrilineally inherited mitochondrial DNA (mtDNA) continues to be a preferred genetic marker for evaluating population structure and phylogeography of marine turtles. Insights from mtDNA studies have informed management of hawksbills in the Atlantic, particularly the Caribbean, and Indo‐Pacific oceans (Broderick et al. 1994; Bass et al. 1996; Okayama et al. 1999; Monzón‐Argüello et al. 2011; LeRoux et al. 2012; Vargas et al. 2015).

Here, we use mtDNA from several hawksbill rookeries in the EP to (1) understand overall genetic diversity in the region, (2) evaluate stock structure and connectivity of hawksbill rookeries, (3) assess what geographic scales of management are appropriate, and (4) examine evolutionary history of the species. This study represents the first genetic characterization of hawksbill rookeries in the data deficient EP Ocean region.

## Methods

### Sample collection and archiving

**Figure 2:**
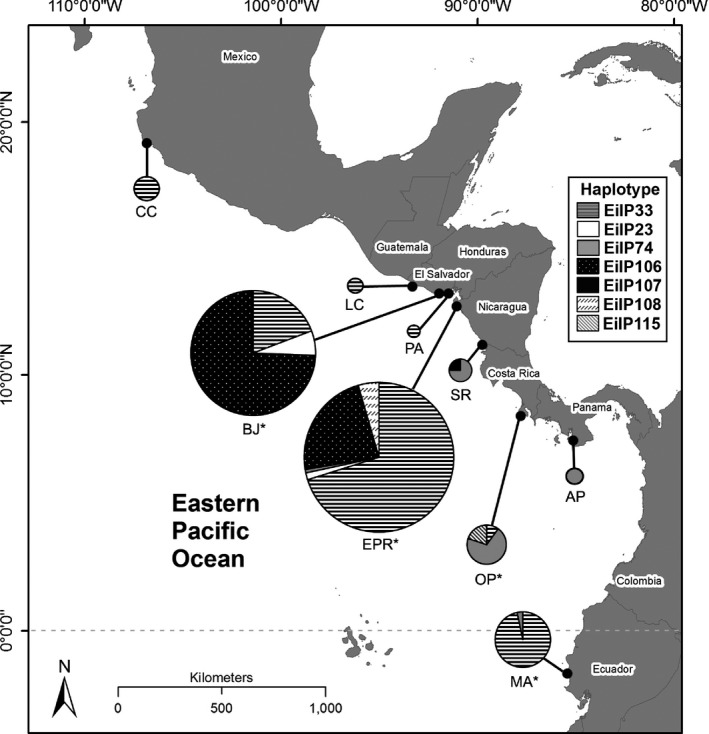
Map of hawksbill sampling locations and corresponding haplotype frequency distributions, with node sizes corresponding to sample sizes for each given site. CC: Costa Careyes, LC: Los Cobanos, BJ: Bahía de Jiquilisco, PA: Punta Amapala, EPR: Estero Padre Ramos, SR: Southern Rivas, OP: Osa Peninsula, AP: Azuero Peninsula, MA: Machalilla. Projection: Mollweide.

Tissue samples were collected at nine hawksbill rookeries spanning the EP Rim (Fig. 2) between 2008 and 2014. These rookeries represent nesting sites located in five countries, including Mexico (classified as North America), El Salvador, Nicaragua, Costa Rica, Panama (collectively classified as Central America) and Ecuador (classified as South America) (Table 1), covering the entire latitudinal nesting extent for the species in the region.

**Table 1:** Hawksbill sample collection country, location, and area (NA = North America; CA = Central America; SA = South America), nesting habitat (OC = open‐coast; ME = mangrove estuary) estimated number (range) of total nesting females at each rookery (Nf) (Gaos et al. 2010; Altamirano 2014; LeMarie et al. 2014; Liles et al. 2015), sample size (n), number of haplotypes (H), nucleotide (*π*), and haplotype (h) diversities with associated standard deviation (SD), and haplotype frequencies. Bolded haplotype nomenclature represents newly identified nesting haplotypes

Location	Area	Latitude	Longitude	Habitat	Nf	*n*	H	*π*	SD	*h*	SD	Haplotype						
												EiIP33	EiIP23	**EiIP74** *	**EiIP106** *	**EiIP107**	**EiIP108**	**EiIP115** *
**Mexico**	**NA**					**8**						**8**	0	0	0	0	0	0
Costa Careyes		19° 26′ N	105° 01′ W	OC	14–18	8	1	–	–	–	–	8	0	0	0	0	0	0
**El Salvador**	**CA**					**81**						**18**	**5**	**0**	**58**	**0**	**0**	**0**
Los Cobanos		13° 32′ N	89° 49′ W	OC	25–30	2	1	–	–	–	–	2	0	0	0	0	0	0
Bahía de Jiquilisco		13° 11′ N	88° 27′ W	ME	140–155	78	3	0.0007	0.0006	0.4113	0.0583	15	5	0	58	0	0	0
Punta Amapala		13° 09′ N	87° 55′ W	OC	10–15	1	1	–	–	–	–	1	0	0	0	0	0	0
**Nicaragua**	**CA**					**138**						**94**	**2**	**4**	**31**	**1**	**6**	**0**
Estero Padre Ramos		12° 46′ N	87° 28′ W	ME	195–210	134	5	0.0006	0.0006	0.4555	0.0416	94	2	1	31	0	6	0
Southern Rivas		11° 07′ N	85° 46′ W	OC	8–10	4	2	–	–	–	–	0	0	3	0	1	0	0
**Costa Rica**	**CA**					**10**						**1**	**0**	**7**	**0**	**0**	**0**	**2**
Osa Peninsula		8° 39′ N	83° 42′ W	OC	16–20	10	3	0.0015	0.0012	0.5111	0.1643	1	0	7	0	0	0	2
**Panama**	**CA**					**2**						**0**	**0**	**2**	**0**	**0**	**0**	**0**
Azuero Peninsula		7° 29′ N	80° 57′ W	OC	4–6	2	1	–	–	–	–	0	0	2	0	0	0	0
**Ecuador**	**SA**					**30**						**29**	**0**	**1**	**0**	**0**	**0**	**0**
Machalilla		1° 33′ S	80° 50′ W	OC	32–36	30	2	0.0001	0.0002	0.0667	0.0613	29	0	1	0	0	0	0
**OVERALL**					**436**–**492**	**269**	**7**	**0.000886**	**0.000751**	**0.5778**	**0.0211**	**150**	**7**	**14**	**89**	**1**	**6**	**2**

*Haplotype not previously identified in a nesting colony.

Monitoring patrols to locate hawksbills were conducted at the project sites and when a female turtle was encountered nesting, tissue samples were collected from the shoulder area (Dutton 1996). In order to allow individual identification, turtles were tagged with PIT (passive integrated transponder) tags and double inconel flipper tags (Style 681, National Band and Tag Company, Kentucky, USA). Additional samples consisted of collecting an entire single flipper from dead hatchlings salvaged from nests, taking care to avoid sampling multiple individuals from nests laid by the same female through flipper tag monitoring. After collection, samples were placed in vials containing >95% ethanol or water saturated with sodium chloride, which were subsequently stored in a −20°C freezer.

### Laboratory procedures

DNA was extracted from hawksbill tissue and prepared for PCR (polymerase chain reaction) (Innis et al. 1990) using either sodium chloride extraction (modified from Miller et al. 1988) or an X‐tractor Gene robot (Corbett Robotics, San Francisco, CA). An ~880‐bp segment of the mtDNA control region d‐loop was amplified using primers LCM‐15382 and H950 g (Abreu‐Grobois et al. 2006; Dutton et al. 2007), which was subsequently trimmed to 766 bp as this region contains optimal, high‐quality reads (LeRoux et al. 2012; Jensen et al. 2013; Dutton et al. 2014). Sequences were amplified by PCR on an ABI 2720 therma cycler (Applied Biosystems, Foster City, CA). PCRs were set up in a 25 *μ*L reaction containing reagents [purified H_2_O (18.25 *μ*L), 10× Mg buffer (2.5 *μ*L), DNTPs (1.5 *μ*L), *Taq* polymerase (0.25 *μ*L), primers (0.75 *μ*L), and template DNA (1 *μ*L)] using the following incubation profile: initial denaturation at 94°C for 2 min, followed by 30 cycles of denaturing at 94°C for 50 sec, primer annealing at 56°C for 50 sec, and primer extension at 72°C for 1 min, followed by a final primer extension for 5 min at 72°C. PCR products were analyzed for quality and quantity on agarose gels and then purified prior to sequencing using ExoSap. Cycle sequencing reactions were conducted with Big Dye fluorescent dye terminator (Applied Biosystems), and the fragments were analyzed using Sanger sequencing on an automated sequencer (Applied Biosystems Inc. model 3730). Sequences were evaluated for both forward and reverse reactions, and all potential new sequences were resequenced to ensure correct identification. We assigned haplotypes by comparing aligned sequences against a local reference library of ~760 bp using Geneious v. R8 (Biomatters Inc.) as well as searching the database on GenBank (http://www.ncbi.nlm.nih.gov) for sequences within our reading frame. New sequences were deposited in GenBank under the following Accession Numbers: KR012503, KR012504, KT003685, KR012505.

### Data analyses

We calculated haplotype (*h*) and nucleotide (*π*) diversities using Arlequin v 3.5.1.2 (Excoffier and Lischer 2010) for four rookeries (Fig. 2) where sample sizes represented a minimum of 50% of the estimated nesting population (both low‐end and high‐end ranges; see Table 1). Pairwise *F*_ST,_ Φ_ST_ and AMOVA (analysis of molecular variance) comparisons were also calculated within Arlequin to test for population structure among these rookeries and the results of these methods were compared. We used the program IBD (Jensen et al. 2005) to conduct a Mantel test of isolation by distance on each population. We performed a chi‐square test (Sokal and Rohlf 1981; Roff and Bentzen 1989) in Microsoft Excel to detect significant shifts in haplotype frequencies between hawksbills nesting in mangrove estuaries versus along open‐coast beaches, the former including samples from Bahía de Jiquilisco and Estero Padre Ramos (pooled *n* = 212), and the latter including samples from Los Cóbanos, Punta Amapala, Southern Rivas, Osa Peninsula, and Azuero Peninsula (pooled *n* = 19).

To provide a temporal estimate of hawksbill lineage divergence, we used a relaxed clock model in BEAST v 1.4.4 (Drummond and Rambaut 2007). The best substitution model of sequence evolution (GTR+G) was determined using Jmodeltest (Posada 2008), and the program was run for 10 million generations with a constant population size tree prior and sampled every 1000 generations. Using calibrations performed with out‐group data from other species for which previously published divergence estimates existed (Naro‐Maciel et al. 2008)(*N. depresses*– GenBank Accession Number U40662, *C. mydas* – GenBank Accession Number JF926560, *C. caretta* – GenBank Accession Number AJ001076, *L. kempii* – GenBank Accession Number AF051777 and *L. olivacea* – GenBank Accession Number AF514311), we estimated time to most recent common ancestry (TMRCA) between EP haplotypes and selected hawksbill haplotypes from the Indo‐Pacific (Vargas et al. 2015; GenBank Accession Numbers KT934050‐KT934101) and Caribbean (LeRoux et al. 2012; GenBank Accession Numbers JN998509, JN998512, JN998521, JN998523, JN998525). Effective sample sizes (ESS > 2000) and 95% highest posterior densities (HPDs) were observed for all parameters in TRACER v1.3 (Rambaut and Drummond 2003). A consensus tree with divergence times was obtained from the 10,000 generated trees, after discarding the first 2500 as burn‐in.

## Results

### Haplotype and nucleotide diversity

A 766‐bp mtDNA control region sequence was aligned for 269 turtles. We identified six polymorphic sites that describe seven haplotypes, two of which have been previously identified at rookeries and five of which have not. Both previously identified nesting haplotypes, EiIP33 and EiIP23 (GenBank Accession Numbers KT934080 & KT934070, respectively), are also found in the Indo‐Pacific, the former being widespread and encountered at numerous rookeries across the region and the latter only found in the Solomon Islands (Vargas et al. 2015). All five of the new nesting haplotypes were encountered solely in Central American rookeries and were not identified in either the North or South American rookeries. Three of the five new nesting haplotypes were previously identified for juvenile hawksbills encountered at foraging grounds located along Pacific Colombia (*n* = 3; EiIP74, EiIP106, and EiIP115; Trujillo‐Arias et al. 2014) and the Howicks Island group in the Great Barrier Reef (*n* = 1; EiIP74; GenBank Accession Number KT964296; Bell et al. unpubl. ms). Haplotype EiIP33 was the most common and widespread haplotype we encountered (55.8% of all samples), followed by EiIP106 (33.1%), EiIP74 (5.2%), EiIP23 (2.6%), EiIP108 (2.2%), EiIP115 (0.7%), and EiIP107 (0.4%). Haplotype EiIP107 was found in a single turtle nesting in Southern Rivas, Nicaragua. Five of the sequence variable sites were transitions and one was a transversion. Haplotype diversities within the four main rookeries ranged from *h *=* *0.0667 to 0.5111, with an overall value of *h *=* *0.5778 (Table 1). Nucleotide diversities ranged from *π *= 0.0001 to 0.0015, with an overall value of *π *= 0.0009 (Table 1).

**Figure 3:**
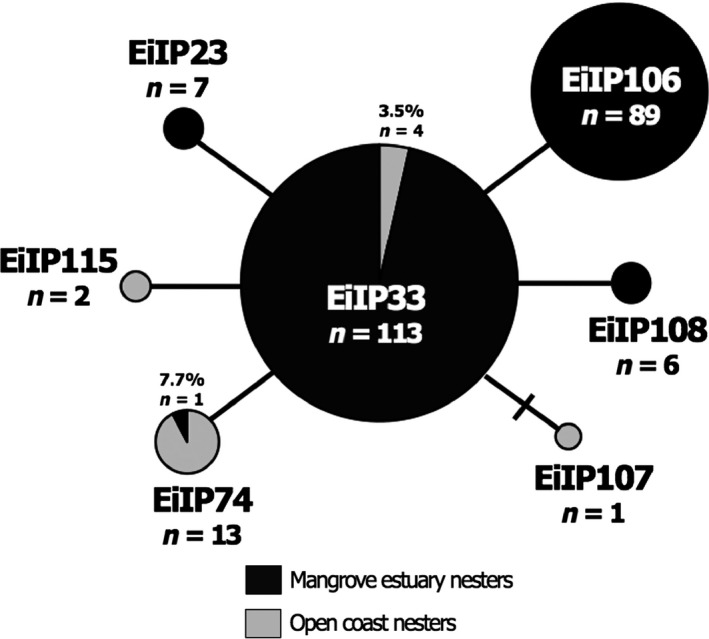
Haplotype network of hawksbills nesting in mangrove estuaries (black) and along open‐coast beaches (gray). The size of the nodes represents the relative frequency of the haplotypes out of the total sample. Vertical and horizontal bars represent one extra mutational step connecting two haplotypes. This haplotype network is specific to samples collected from Central American rookeries, but with the exception of sample size, is representative of all rookeries included in this study.

The most parsimonious median‐joining network of haplotype sequences indicated that all EP hawksbill turtles represent one marginally divergent phylogroup with each haplotype separated by only one substitution, with the exception of EiIP107, which was separated by two substitutions (Fig. 3). EiIP33 represented the basal haplotype in this network, suggesting it might be ancestral to all other haplotypes.

### Interpopulation analysis and nesting habitat analysis

*F*_ST,_ Φ_ST_, and the AMOVA showed highly significant (*P* < 0.001) structure among the four main rookeries included in our interpopulation analysis (Table 2). Because results from these tests were the same, we used only *F*_ST_ for all subsequent analyses. Mantel test results indicated no significant relationship between genetic and geographic distance, and thus, distance appears to not be a predictor of genetic divergence in EP hawksbills. Haplotypes found among hawksbills nesting in mangrove estuaries were rarely found in those nesting along the open coast in Central America and vice versa (Fig. 3). The haplotype frequency differences between these two groups were highly significant (*P* = 0.0000, Chi = 167.66, df = 6). Haplotypes EiIP106 and EiIP108 were only found in hawksbills nesting in mangrove estuaries.

**Table 2:** *F*_ST_ values (with associated *P*‐values) and distances (km) among primary hawksbill nesting rookeries

	Bahía de Jiqulisco	Estero Padre Ramos	Osa Peninsula	Machalilla
Bahía de Jiquilisco	–	115 km	750 km	1835 km
Estero Padre Ramos	0.3706**	–	645 km	1745 km
Osa Peninsula	0.5541**	0.4916**	–	1115 km
Machalilla	0.6433**	0.1257*	0.7791**	–

*P <* *0.05, **P <* *0.0005.

### Phylogenetic analysis

**Figure 4:**
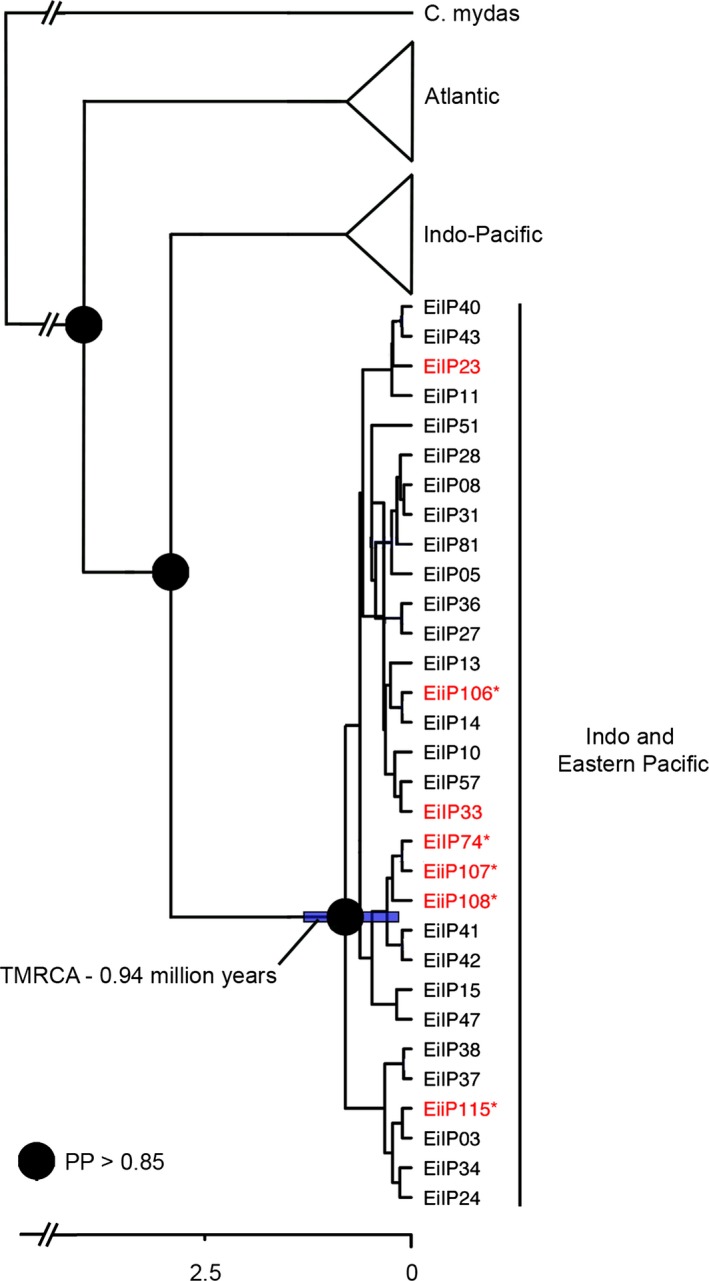
Phylogeny of hawksbill haplotypes in the EP (red font), Indo‐Pacific, and Atlantic (Caribbean) oceans (LeRoux et al. 2012; Vargas et al. 2015). Mean HPD value estimated for the tree node for the clade that includes samples from the Indo and Eastern Pacific are indicated together with its corresponding 95% HPD intervals (blue shaded horizontal bar). Asterisk indicates haplotype is unique to rookeries in the Eastern Pacific. TMRCA million years before present shown on the *x*‐axis.

As has been previously documented, phylogenetic analysis revealed strong node support for a deep bifurcation between lineages in the Atlantic and the Pacific (Okayama et al. 1999; Vargas et al. 2015). We found that EP hawksbills group in a clade with individuals from the Indo‐Pacific oceans, rather than those in the Atlantic (Fig. 4). The phylogenetic split for the all haplotypes found in the EP revealed a divergence date from Bayesian estimates of about 0.94 mya (95% HPD: 0.2977–1.445; Fig. 3).

## Discussion

The results from our mtDNA analysis provide resolution on the genetics of the recently “rediscovered” hawksbill rookeries in the EP and give us an opportunity to compare patterns of genetic diversity, differentiation, and phylogeography of the species. In doing so, we obtain a more complete picture of the life history of hawksbills in the EP, with important implications for conservation and management.

### Genetic diversity and stock structure

The average haplotype and nucleotide diversity values across the four principal hawksbill rookeries in the EP (*h *=* *0.3612 and *π *= 0.0007, respectively) are lower than the average values found for rookeries in the Indo‐Pacific (*n* = 11; *h *=* *0.5863, *π *= 0.0086; Vargas et al. 2015) and Caribbean (*n* = 13; *h *=* *0.396, *π *= 0.0101; LeRoux et al. 2012). We identified only seven nesting haplotypes across EP rookeries, which span >3000 km, compared to 52 in the Indo‐Pacific (Vargas et al. 2015) and 23 in the Caribbean (LeRoux et al. 2012; Monzón‐Argüello et al. 2011), which span approximately 11,000 km and 3000 km, respectively. In contrast to these other ocean regions, hawksbill rookeries in the EP are characterized by highly similar haplotypes, all falling within a single clade and separated by only one or two base pair substitutions (Figs. 3 and 4). The presence of a greater number of rookeries, higher overall diversity, as well as a greater number of haplotypes that are substantially more divergent in the Indo‐Pacific and Caribbean (LeRoux et al. 2012; Vargas et al. 2015) suggests hawksbill populations in these regions have been larger and more stable over time compared to those in the EP. Indeed, the low genetic diversity in the EP is not surprising considering that the region hosts multiple small rookeries. Five of the nine rookeries are estimated to be comprised of <20 individual nesting females, and the two largest rookeries in Bahia de Jiquilisco and Estero Padre Ramos are estimated to host a maximum of only 155 and 210 nesting females, respectively (Gaos et al. 2010; Altamirano 2014; Liles et al. 2015; Table 1). Low genetic diversity could also be the result of relatively recent colonization of the EP (see *Evolutionary history and phylogeography*), leading to a genetic bottleneck.

Despite low genetic diversity, our results indicate high levels of mtDNA differentiation between hawksbill rookeries in Bahía de Jiquilisco, Estero Padre Ramos, Osa Peninsula, and Machalilla (Table 1). The Bahía de Jiquilisco and Estero Padre Ramos rookeries are separated by only 115 km; yet, we still found strong genetic structure despite their proximity. These results are robust as our largest sample sizes are from these two rookeries (*n* = 78 and *n* = 134, respectively). While previous satellite telemetry has tracked postnesting females from both of these rookeries to foraging grounds in Bahía de Jiquilisco (Gaos et al. 2012b), our results indicate that natal homing maintains segregation of female nesting populations even at the scale of 115 km. Strong nesting site fidelity by females is further corroborated by ongoing monitoring projects at these two sites, which have never observed a single turtle nesting at both locales (Altamirano 2014; Liles et al. 2015).

We found five haplotypes that appear exclusive to rookeries in the EP and these haplotypes were only found in Central America. While our sample sizes are representative of most of the rookeries, logistical limitations with attaining samples from some of the minor rookeries (e.g., Los Cobanos in El Salvador), as well as the potential discovery of new rookeries, may reveal that additional haplotypes do exist. Nonetheless, considering that our sampling sites represent the primary known rookeries in the EP and that we attained samples from approximately 50% of the nesting females estimated to remain in the region (Gaos et al. 2010; Altamirano 2014; Liles et al. 2015), additional haplotypes are likely to be rare. Notwithstanding this perspective, additional rare haplotypes may have local importance if found at high frequency in poorly sampled areas. Ongoing work currently occurring at major and minor nesting sites will allow for future verification.

### Evolutionary history and phylogeography

From a phylogeographic perspective, there are two possibilities of how hawksbills arrived to the EP: either they radiated out of the Indo‐Pacific at some time in the past or they represent a relict population isolated from the Atlantic by the rising of the Panama Isthmus (Bowen and Karl 2007). There is evidence to support both of these distribution pathways in various fish and invertebrate species (Collin 2003; Alejandrino and Valdes 2006; Lessios and Robertson 2006; Whitney et al. 2012; Cowman and Bellwood 2013). Although the 7000+ km expanse of deep water separating the Indo‐Pacific and EP, often referred to as the ‘Eastern Pacific Barrier’, regularly restricts dispersal of marine organisms (Lessios and Robertson 2006), our phylogenetic analysis indicates hawksbills in the EP are more closely related to individuals from the Indo‐Pacific (Fig. 4), providing strong evidence that hawksbills radiated across the Eastern Pacific Barrier. This finding concurs with previous research on leatherback (*Dermochelys coriacea*), olive ridley (*Lepidochelys olivacea*), and green turtles (*Chelonia mydas*), which has found populations of each species in the EP to be more closely related to conspecifics in the Indo‐Pacific than to those in the Atlantic (Bowen & Karl 1999; Bowen et al. 1998; Dutton et al. 1999, 2013).

Furthermore, the low genetic diversity and star‐shaped haplotype network (Fig. 3) for hawksbills in the EP suggest the species likely radiated out of the Indo‐Pacific on a relatively recent evolutionary timescale (Gillespie 1984). We also documented the first sharing of a nesting haplotype across the entire expanse of multiple ocean basins, with haplotype EiIP33 (not found in the Atlantic) spanning from the westernmost Indo‐Pacific Ocean (i.e., Indian Ocean) to the EP Ocean (Tabib et al. 2011; Vargas et al. 2015; this study). The presence of this haplotype in nesting populations across these ocean basins further supports a recent radiation. Haplotype EiIP23 has also been documented (n=2) at a rookery on the Solomon Islands (Vargas et al. 2015), and this may suggest multiple colonization events from the west, or more likely, the presence of this haplotype in both regions is the result of homplasy. An additional line of research related to colonization history involves small hawksbill rookeries on the Hawaiian archipelago and several other islands in the central Pacific (Balazs 1978, 1984; Van Houtan et al. 2012), which may have served as pathways to colonization of the EP as has been postulated for green turtles (Dutton et al. 2014). Genetic characterization of these rookeries will help elucidate this possibility.

Recent satellite telemetry tracking research found that adult hawksbills in the EP tend to undertake short migrations and spend the majority of their lives in shallow, highly neritic habitats (Gaos et al. 2012b,c). However, the movement and dispersal of hatchlings remains much less clear and it is widely accepted that as passive drifters, hatchlings can make transoceanic migrations as they become entrained in large ocean currents, often across major ocean barriers (Bolten 2003; Blumenthal et al. 2009; Putman and He 2013). Transoceanic migrations for hatchlings have been postulated from west to east in the Pacific Ocean for loggerhead turtles (*Caretta caretta*) (Bowen et al. 1995; Boyle et al. 2009), and it is possible that hawksbill hatchlings undertake a similar migration. However, the presence of the EiIP33 and EiIP23 nesting haplotypes throughout the Pacific Ocean basin complicates discernment of rookery origin (e.g., using mixed stock analysis). This lack of resolution will also be problematic for precise mtDNA stock assignments for foraging grounds and fisheries bycatch in the region (Dutton et al. 2014).

Interestingly, the only two previous records of haplotype EiIP74, which we identified in multiple Central American rookeries, came from two foraging animals, one encountered along Pacific Colombia (Trujillo‐Arias et al. 2014) and the other at the Howicks Island group in the Great Barrier Reef, Australia (Bell et al.). Barring discovery of nesting rookeries in other parts of the Pacific that harbor the EiIP74 haplotype, our findings suggest that hatchlings are able to emigrate out of the EP (e.g., into Australian waters). Westerly hatchling dispersal from Central America could be facilitated via either the North or South Equatorial currents in conjunction with the California and Peru (or Humboldt) currents, respectively.

We also found potential evidence for fine scale genetic segregation between hawksbills nesting in mangrove estuaries and along open‐coast beaches in Central America: The haplotypes found in turtles nesting in mangrove estuaries (at Bahía de Jiquilisco and Estero Padre Ramos) were rarely present in open‐coast nesters (at Los Cobanos, Punta Amapala, Southern Rivas, Osa Peninsula and Azuero Peninsula) and vice versa (Fig. 3). While such an inference must take into consideration the small sample size from open‐coast beaches (*n* = 19) from five rookeries, our preliminary results suggesting there may be mtDNA genetic differences between hawksbill nesting in these two habitats are compelling.

Regardless of population differences, considering that haplotypes EiIP106 and EiIP108 are unique to hawksbills that nest in mangrove estuaries and that this behavior appears exclusive to hawksbills along Pacific Central America (Gaos et al. 2012a; Liles et al. 2015), we assert the existence of a novel mangrove estuary “reproductive ecotype” (Taylor et al. 1997) in the region. While mangrove estuarine habitats are present in North and South America, and adult hawksbills have been documented establishing foraging home ranges in these habitats in the latter region (Gaos et al. 2012a,b), mangrove estuary nesting does not appear to occur in these areas and unique haplotypes have yet to be identified. Hawksbills in the Central American rookeries likely began exploiting inshore mangrove estuaries for foraging due to a lack of coral reefs in the region (Glynn 1997; Gaos et al. 2012a), which probably eventually led to nesting in these areas as well. It remains unclear whether the unique mtDNA haplotypes for hawksbills nesting in these habitats is the result of preferential selection by turtles or simply the result of a geographic survivorship effect (sensu van Dam et al. 2008). In the latter scenario, being less conspicuous in mangrove estuaries, hawksbills are less susceptible to overexploitation, and thus, individuals from these habitats have gradually comprised a greater proportion of the population. This would also explain why mangrove rookeries harbor a larger proportion of the genetic diversity in the region.

The Central American rookeries have also likely been more stable over time compared to those in North and South America, which represent the latitudinal nesting extremes for the species. Fossil records of the Early Pleistocene, which roughly correspond to our TMRCA estimates, show that glacial conditions had little effect on the fauna at low latitudes, with stable conditions maintained in equatorial regions (e.g., Central America) (see refs in Encalada et al. 1996). Climatic stability in the tropics during times of both glacial minima and maxima would likely allow hawksbill nesting populations in Central America to persist, while during glacial maxima rookeries located at geographic extremes would have more difficulty persisting. The fact that hawksbill genetic diversity is location‐specific in the EP is likely influenced contemporaneously by a number of factors, including colonization history, climate regimes, population size, gene flow among rookeries, and human exploitation (Fisher 1930; Wright 1931; Mortimer and Donnelly 2008).

### Conservation implications

Successfully managing a marine turtle population that was until recently considered virtually absent in the EP requires the establishment of baseline information, including the genetic data we outline here. MUs (Management units sensu Moritz 1994) are designed to protect nesting populations that are demographically and genetically independent and that would not be recolonized on ecological (versus evolutionary) timeframes that are relevant to wildlife managers (Waples 1991, 1995; Avise 1995; Encalada et al. 1996). MUs are largely defined by genetic metrics based on the premise that genetic diversity protects species against extinction by providing the potential for phenotypic adaptability (Moritz 1994), which can help buffer against environmental perturbations. For marine turtles, past MUs have been largely based on mtDNA findings that show signs of significant population differentiation (Dutton et al. 1999, 2014; Watanabe et al. 2011; Jensen et al. 2013). Hawksbills in the EP have previously been referred to as a single population, primarily due to the paucity of hawksbill data from the region. Based on our mtDNA results, the four principal nesting rookeries highlighted in this study each merit recognition as individual MUs and demonstrate the existence of at least four distinct populations. It is important these MUs be incorporated into national and international management strategies and that conservation efforts focus on the viability of hawksbill populations at each of these sites. Nesting rookeries for which limited samples have been collected (e.g., Costa Careyes, Los Cobanos and Punta Amapala) or where sampling has yet to be initiated (e.g., Aserradores in Nicaragua and Playa Rosada in Ecuador) may reveal that the delineation of additional MUs is warranted. While large geographic distances are sometimes used as criteria for delineating MUs (Moritz 1984), our genetic findings show that proximate rookeries can be genetically distinct and in such cases, classifying these populations as a single MU would be misguided.

Anthropogenic‐induced global warming is predicted to lead to more extreme climactic events and an overall increase in average global temperatures (IPCC 2013), which may negatively impact the ability of hawksbill populations to grow and/or recolonize on both ecological and evolutionary timescales, heightening the need to protect extant nesting colonies (Mora et al. 2007). This includes smaller (e.g., <20 females/year) and more isolated nesting colonies, which are at greater risk of extinction, particularly in the face of rapidly shifting environmental conditions (Jules and Shahani 2003). However, conserving larger nesting colonies (e.g., Bahía de Jiquilisco and Estero Padre Ramos) is key as these rookeries may provide “storage effects” (Warner and Chesson 1985), in which via one or two solid recruitment classes per generation they may enable persistence of the species on ecological timescales. Furthermore, on evolutionary timescales these rookeries may serve as the primary source of females for re‐colonization of the entire EP. Protecting both the open‐coast and mangrove estuary nesting beaches in the EP appears to be a critical safeguard for genetic diversity of the species.

### Future directions

With the escalating number of individuals and institutions embarking on marine turtle (including hawksbill) genetic studies, particularly those implementing mtDNA markers, the need to regiment sequence identification and nomenclature is heightened. Increased communication, collaboration, and data sharing among researchers are among the most effective mechanisms to ensure the integrity of genetic studies. In this respect, the Atlantic‐Mediterranean loggerhead genetics working group (Shamblin et al. 2014) and the online mtDNA sequence database managed by the Archie Carr Center for Sea Turtle Research (http://accstr.ufl.edu/resources/mtdna-sequences/) represent prime examples of collaboration and information sharing on marine turtle genetics. Efforts to expand and/or reproduce these models for other species and/or regions would be prudent.

Future genetic studies using nDNA (nuclear DNA) markers (e.g., microsatellites, SNPs) are needed to verify and further evaluate the genetic characterization of hawksbills in the EP as the data from mtDNA markers only reflect variation among female lineages (Carreras et al. 2007; Bowen and Karl 2007). Among other advantages, the use of nDNA markers may help further define population structure, understand overall genetic diversity, and elucidate sex‐biased gene flow (Bowen and Karl 2007). Recent research by Dutton et al. (2013) using 17 microsatellite loci detected population structure in leatherback turtles that was indistinguishable with mtDNA data, highlighting the relevance of including nDNA markers for evaluations of this taxon. It is important to recognize that the results from mtDNA and nDNA markers do not represent conflicting schemes, but rather reflect geographic structuring differences on distinct DNA lineages (Bowen 2007), which together can provide a more complete understanding of genetic structure.

## Conflict of Interest

None declared.
